# Functional and Expression Studies of iPSC-Derived Cardiomyocytes Carrying a Novel HCM-Associated *MYPN* Genetic Variant

**DOI:** 10.3390/genes17040456

**Published:** 2026-04-14

**Authors:** Elena V. Dementyeva, Ekaterina S. Klimenko, Margarita Y. Sorokina, Anastasia K. Zaytseva, Maxim T. Ri, Ekaterina G. Nikitina, Dmitriy A. Kudlay, Anna M. Zlotina, Svetlana I. Tarnovskaya, Yuri V. Vyatkin, Dmitriy N. Shtokalo, Suren M. Zakian, Anna A. Kostareva

**Affiliations:** 1 Institute of Cytology and Genetics, Siberian Branch of the Russian Academy of Sciences, 630090 Novosibirsk, Russia; dementyeva@bionet.nsc.ru (E.V.D.); zakian@bionet.nsc.ru (S.M.Z.); 2 Institute of Chemical Biology and Fundamental Medicine, Siberian Branch of the Russian Academy of Sciences, 630090 Novosibirsk, Russia; 3 Almazov National Medical Research Centre, Institute of Molecular Biology and Genetics, 197341 Saint-Petersburg, Russiasorokina_my@almazovcentre.ru (M.Y.S.);; 4 The Center for Molecular and Cellular Biology, 121205 Moscow, Russia; 5 Department of Pharmacognosy and Industrial Pharmacy, Faculty of Fundamental Medicine, Lomonosov Moscow State University, 119991 Moscow, Russia; 6 Department of Pharmacology at the Institute of Pharmacy, Sechenov First Moscow State Medical University (Sechenov University), 119048 Moscow, Russia; 7 NRC Institute of Immunology FMBA of Russia, 115522 Moscow, Russia; 8 Sechenov Institute of Evolutionary Physiology & Biochemistry, Russian Academy of Sciences, 194223 St. Petersburg, Russia; 9 Novel Software Systems, 630090 Novosibirsk, Russia; vyatkin@gmail.com; 10 AcademGene LLC, 630090 Novosibirsk, Russia; 11 A.P. Ershov Institute of Informatics Systems, Siberian Branch of the Russian Academy of Sciences, 630090 Novosibirsk, Russia; 12 Department of Women’s and Children’s Health, Karolinska Institutet, 17176 Stockholm, Sweden

**Keywords:** myopalladin, hypertrophic cardiomyopathy, genetic variants, induced pluripotent stem cells, cardiomyocytes

## Abstract

**Background/Objectives:** Variants of *MYPN*, encoding a sarcomeric protein myopalladin, are associated with different types of cardiomyopathies and myopathies. However, the molecular mechanisms of *MYPN*-associated pathologies are still poorly understood. **Methods:** In this study, we generated induced pluripotent stem cells (iPSCs) from a hypertrophic cardiomyopathy patient carrying a novel p.N989I (c.2966A>T) variant of *MYPN* and used iPSC-derived cardiomyocytes to examine the impact of the variant on biophysical characteristics and transcriptomic profile. **Results:** No significant changes in parameters of calcium transients, sodium current and action potential were found in iPSC-derived cardiomyocytes with the p.N989I (c.2966A>T) variant of *MYPN* compared to non-isogenic cells from an unrelated healthy donor. At the transcriptomic level, *MYPN*-N989I cardiomyocytes demonstrated an upregulation of genes linked to cell cycle, mitotic spindle, microtubule cytoskeleton organization, and myogenic program genes. Downregulation of sarcomeric, Z-disc- and cell junction-associated genes, as well as genes involved in ATP synthesis, oxidative phosphorylation, and the SRF-signaling pathway, was also revealed. **Conclusions:** Our data suggest that the p.N989I (c.2966A>T) variant of *MYPN* plays a dual role in hypertrophic cardiomyopathy pathogenesis, disrupting not only sarcomeric and cytoskeletal organization but also the regulation of the muscle gene program.

## 1. Introduction

Myopalladin, encoded by the *MYPN* gene, was identified over twenty years ago as one of the key α-actinin-binding proteins involved in actin cytoskeleton organization, polymerization, and stability [1]. Based on its homology with the earlier-described palladin and myotillin, myopalladin was shown to play an important role in actin filament assembly of both sarcomeric and cytoskeletal pools of F-actin in muscle cells. As a striated muscle-specific protein, it is localized in the I-band and Z-line of the sarcomeric structure and interacts with several structural components of the Z-disc, such as nebulette and titin. Myopalladin is also capable of interacting with nucleus-translocating proteins of the Z-disc area, including CARP and ANKRD2. The latter observation suggests a mechanotransduction function of myopalladin, positioning it as an important regulator of contractile strength and relaxation through the initiation of several gene expression programs. The ability of myopalladin to provide a structural integrity of the Z-disc [2] and to translocate to the nucleus together with CARP makes it a potential regulator of myogenic gene program via interaction with MRTF-A, thereby regulating SRF signaling and cell cycle-related genes [3]. In addition, *MYPN*-knockout mice demonstrate the alteration in several signaling pathways and kinase expression, such as mitogen-activated protein kinases Erk1/2, JNK, PAK1, as well as Akt-phosphorylation [3]. All together, these findings confirm the important role of *MYPN* in Z-line architecture and stress-detecting signaling of striated muscle cells.

As a Z-line-associated protein of the sarcomere, *MYPN* has attracted attention as a possible cardiomyopathy- and myopathy-causing gene. Similar to other Z-line proteins, myopalladin has been described in connection to several types of cardiomyopathies—hypertrophic (HCM) [4,5], dilated (DCM) [6,7,8], restrictive (RCM), and left-ventricular non-compaction [9]. It was also detected as a causative gene for slow progressive congenital cap myopathy—a subtype of nemaline myopathy—with autosomal recessive inheritance [10].

Previously, we described an HCM patient carrying a novel p.N989I (c.2966A>T) variant of *MYPN* [11]. This variant was detected using clinical exome sequencing and was the only one identified among HCM-related genes. The variant carrier was a female diagnosed with non-familial obstructive HCM after 60 years of age according to standard HCM criteria, and her interventricular septum thickness was 25 mm. The substitution is localized to the alpha-actinin-interacting region of the protein [1] and exhibits pathogenic properties according to in silico analysis data. However, the clinical significance of the variant and possible mechanisms underlying its pathogenicity remain unknown. According to ACMG classification, this variant can currently be interpreted only as a variant of unknown clinical significance. Generating induced pluripotent stem cells (iPSCs) from HCM patients and their subsequent differentiation into cardiomyocytes has been successfully used to create cell models for unraveling pathogenetic mechanisms of the disease [12,13,14,15,16,17,18,19,20,21,22]. In this study, we applied the iPSC-based approach to generate the first cell model for HCM caused by a *MYPN* variant to further uncover its functional and potential clinical role. To decipher the role of *MYPN* and the p.N989I (c.2966A>T) variant in HCM pathogenesis, we analyzed the functional properties (characteristics of calcium transients, sodium currents, and action potentials) and transcriptome profile of iPSC-derived cardiomyocytes.

## 2. Materials and Methods

### 2.1. Sequence and Structural Analysis of Human Myopalladin and Variant Classification

The sequence for the human myopalladin was obtained from the UniProtKB database under the (https://www.uniprot.org) entry Q86TC9. Domain organization of the protein was performed using the InterPro database (https://www.ebi.ac.uk/interpro/, accessed on 19 August 2025) [23] and lollipops tool (https://joiningdata.com/lollipops/index.html, accessed on 19 August 2025) [24]. Related protein sequences from other organisms for myopalladin were identified by BLASTP [25] search against the Uniref90 database. Similar Ig regions in other human proteins were identified using the human proteome (UniProt ID: UP000005640). Multiple sequence alignment was calculated by Clustal Omega [26] and visualized in Jalview 2.11.5 [27]. The 3D structure of the Ig-like domain of myopalladin (PDB: 2LQR, chain A) [28] was extracted from the Protein Data Bank [29,30]. Variant p.N989I was mapped on the structure according to the sequence alignment. In silico visualization was conducted using the PyMol 2.5 software. The pathogenicity of variant p.N989I was assessed based on the ClinPred and AlphaMissense prediction scores obtained from the dbNSFP database version 4.5 [31].

### 2.2. iPSC Generation

In total, 8.1 × 10^5^ of mononuclear cells (MNCs) of the HCM patient carrying the p.N989I (c.2966A>T) variant of *MYPN* were electroporated with episomal vectors (0.5 μg each, Addgene IDs #41855-41858, #41813-41814) using a Neon Transfection System (Thermo Fisher Scientific, Waltham, MA, USA) program: 1650 V, 10 ms, 3 times. All reprogramming steps were performed according to the Epi5™ Episomal iPSC Reprogramming Kit user guide (Thermo Fisher Scientific, Waltham, MA, USA). Primary iPSC colonies were picked manually, transferred to a feeder layer, and maintained in KnockOut DMEM supplemented with 15% KnockOut Serum Replacement (KoSR), 0.1 mM Non-Essential Amino Acid (NEAA), 1× penicillin–streptomycin, 1 mM GlutaMAX (all Thermo Fisher Scientific, Waltham, MA, USA), 0.05 mM 2-mercaptoethanol (Amresco, Solon, OH, USA), and 10 ng/mL bFGF (SCI-store, Moscow, Russia). Cells were cultured at 37 °C in 5% CO_2_ and passaged at a ratio of 1:10 every 3–5 days with TrypLE™ Express Enzyme (Thermo Fisher Scientific, Waltham, MA, USA).

### 2.3. Mycoplasma and Episome Detection

Mycoplasma contamination and episome elimination in iPSC lines were detected by PCR on a T100 Thermal Cycler (Bio-Rad, Hercules, CA, USA) with BioMaster HS-Taq PCR-Color (2×) (Biolabmix, Novosibirsk, Russia). Programs: 95 °C—3 min; 35 cycles: 95 °C—15 s, 67 °C—15 s, 72 °C—20 s; 72 °C—5 min (mycoplasma) and 95 °C—5 min; 35 cycles: 95 °C—15 s, 62 °C—15 s, 72 °C—15 s; 72 °C—5 min (episomes). The primers used are given in Table S1.

### 2.4. Spontaneous In Vitro Differentiation

iPSCs were dissociated with 0.15% Collagenase Type IV (Thermo Fisher Scientific, Waltham, MA, USA), transferred onto dishes coated with 1% agarose, and cultivated for 14 days in DMEM/F12 (1:1) medium supplemented with 15% KoSR, 0.1 mM NEAA, 1× penicillin–streptomycin, 1 mM GlutaMAX (all Thermo Fisher Scientific, Waltham, MA, USA). The embryoid bodies formed were plated onto Matrigel (Corning, New York, NY, USA)-coated 8-well Chambered Coverglass (Thermo Fisher Scientific, Waltham, MA, USA) for an additional seven days.

### 2.5. Immunofluorescent Staining

Cells were fixed in 4% paraformaldehyde (Sigma-Aldrich, Darmstadt, Germany) for 10 min, permeabilized in 0.4% Triton-X100 (Sigma-Aldrich, Darmstadt, Germany) for 10 min, and incubated with 1% BSA (VWR, Solon, OH, USA) for 30 min. Cells were incubated overnight at 4 °C with primary antibodies and 1 h at room temperature with secondary antibodies. The antibodies used are listed in Table S1. After each incubation with antibodies, the cells were washed twice for 15 min with PBS. Nuclei were counterstained with DAPI (Sigma-Aldrich, Darmstadt, Germany). Staining was analyzed using a Nikon Eclipse Ti-E microscope and NIS Elements software (ver. 4.51.01) (Nikon, Tokyo, Japan) or Observer.Z1 and ZEISS Efficient Navigation (ZEN) (Zeiss, Oberkochen, Germany).

### 2.6. RT-qPCR

RNA was isolated with TRIzol Reagent (Thermo Fisher Scientific, Waltham, MA, USA) according to the manufacturer’s protocol. Reverse transcription of 1–2 µg of RNA was performed with the M-MuLV reverse transcriptase (Biolabmix, Novosibirsk, Russia). RT-qPCR was carried out on a LightCycler 480 System (Roche, Basel, Switzerland) with BioMaster HS-qPCR SYBR Blue 2× (Biolabmix, Novosibirsk, Russia). Program: 95 °C—5 min; 40 cycles: 95 °C—10 s, 60 °C—1 min. CT values were normalized to *ACTB* using ΔΔCT method. The primers used are given in Table S1.

### 2.7. Karyotyping

Colcemid treatment, cell hypotony and fixation were carried out as described previously [32]. Karyotype was analyzed at the Tomsk National Research Medical Center of the Russian Academy of Sciences using a Zeiss Axio Scope.A1 microscope (Zeiss, Oberkochen, Germany) and Ikaros KARyOtyping (ver.5.5) Software (MetaSystems, Altlussheim, Germany).

### 2.8. Analysis of the Patient-Specific Variant

Exon 14 of *MYPN* was amplified by PCR on a T100 Thermal Cycler (Bio-Rad, Hercules, CA, USA) with BioMaster HS-Taq PCR-Color (2×) (Biolabmix, Novosibirsk, Russia). Program: 95 °C—5 min; 35 cycles: 95 °C—30 s, 60 °C—30 s, 72 °C—30 s; 72 °C—5 min. The primers used are given in Table S1. Sanger sequencing was performed using a Big Dye Terminator V. 3.1. Cycle Sequencing Kit (Applied Biosystems, Austin, TX, USA) and analyzed at the SB RAS Genomics Core Facility.

### 2.9. STR Analysis

STR analysis of the patient-specific iPSC lines and MNCs was performed by Genoanalytica (https://www.genoanalytica.ru) using COrDIS EXPERT 26 Kit (Gordiz, Moscow, Russia) on a 3130 Genetic Analyzer (HITACHI, Applied Biosystems, Tokyo, Japan).

### 2.10. Directed iPSC Differentiation into Cardiomyocytes

iPSCs were cultivated for 2–3 passages in feeder-free conditions in Essential 8 Medium (Thermo Fisher Scientific, Waltham, MA, USA) on wells coated with Geltrex LDEV-Free hESC-Qualified Reduced Growth Factor Basement Membrane Matrix (Thermo Fisher Scientific, Waltham, MA, USA). iPSCs were dissociated with ReLeSR (STEMCELL Technologies, Vancouver, BC, Canada) and plated at a ratio of 1:5–1:10 on 12-well plates. After cells reached 80–90% confluency, differentiation was induced by adding for 48 h RPMI 1640 medium supplemented with 1× penicillin–streptomycin, 1× B27 Supplement minus insulin (all Thermo Fisher Scientific, Waltham, MA, USA) and 6 µM CHIR99021 (Selleckchem, Houston, TX, USA). Then, 72 h after differentiation onset, RPMI 1640 medium supplemented with 1× penicillin–streptomycin, 1× B27 Supplement minus insulin and 5 µM IWR1 (STEMCELL Technologies, Vancouver, BC, Canada) was added for another 48 h. Starting from day 7 of differentiation, cells were cultivated in RPMI 1640 medium supplemented with 1× penicillin–streptomycin and 1× B27 Supplement (Thermo Fisher Scientific, Waltham, MA, USA).

On day 9 of differentiation, cells were subjected to metabolic selection for 6 days in RPMI 1640 medium without D-glucose (Thermo Fisher Scientific, Waltham, MA, USA) supplemented with 1× penicillin–streptomycin, 213 µg/mL L-ascorbic acid 2-phosphate sesquimagnesium salt hydrate, 500 µg/mL bovine serum albumin and 5 mM sodium DL-lactate (all Sigma-Aldrich, Darmstadt, Germany). Cardiomyocytes were dissociated with 2.5× TrypLE™ Select Enzyme (Thermo Fisher Scientific, Waltham, MA, USA), plated on Geltrex-coated 6-well plates and cultivated for two weeks in RPMI 1640 medium supplemented with 1× penicillin–streptomycin, 1× B27 Supplement and 2 µM CHIR99021. On day 30 of differentiation, cardiomyocytes were plated on Geltrex-coated 12-well plates at a ratio of 9 × 10^5^–1 × 10^6^ cells per well. Cells were cultivated for two weeks in the medium for cardiomyocyte maturation [33]—DMEM without D-glucose and L-glutamine (Thermo Fisher Scientific, Waltham, MA, USA) supplemented with 3 mM glucose, 10 mM L-lactate, 5 µg/mL Vitamin B12, 0.82 µM Biotin, 5 mM Creatine monohydrate, 2 mM Taurine, 2 mM L-carnitine, 0.5 mM L-ascorbic acid 2-phosphate sesquimagnesium salt hydrate (all Sigma-Aldrich, Darmstadt, Germany), 0.1 mM NEAA, 1× B27 Supplement, 1% KoSR, 1× penicillin-streptomycin, 2 mM L-glutamine and 0.5% (*w*/*v*) Albumax (all Thermo Fisher Scientific, Waltham, MA, USA).

### 2.11. Calcium Dynamics with Stimulation

Registration of calcium transients was performed with electrical stimulation using IonOptix setup (IonOptix, Westwood, MA, USA). Calcium measurements were registered using calcium-sensitive fluorescent dye Fura-2AM (Thermo Fisher Scientific, Waltham, MA, USA) and analyzed values constituted fluorescence ratio between Fura-2AM channels (binding calcium or not). Cells were seeded on Geltrex-treated 170 µm thick coverglasses (Thermo Fisher Scientific, Waltham, MA, USA), pre-washed with PBS to remove the medium and incubated with 2 μM Fura-2AM in calcium-containing buffer (145 mM NaCl, 4.5 mM KCl, 2 mM MgCl_2_, 10 mM HEPES; 2 mM CaCl_2_) for 40 min prior to imaging. Cells were paced at 0.2 Hz with a 10 V square pulse using a MyoPacer field stimulator (IonOptix, Westwood, MA, USA). The images for each cell were recorded over 2–3 min and the transients for one cell were pooled and averaged. Data was recorded from each dish for no more than 30 min to avoid cell death.

### 2.12. Electrophysiology

Sodium currents and action potentials were recorded using whole-cell patch-clamp configuration at room temperature. For patch-clamp measurements, single cardiomyocytes were seeded on Geltrex-coated coverslips. We used the solutions described earlier [34] with a slight modification in the extracellular solution (50 mM NaCl and 90 mM CsCl instead of 140 mM NaCl).

Microelectrodes were manufactured using a puller (P-1000, Sutter Instrument, Novato, CA, USA). The electrode resistance ranged from 2.0 to 3.5 MΩ. Data acquisition and junction potential correction were done using Axopatch 200B amplifier and Clampfit software version 10.3 (Molecular Devices, San Jose, CA, USA). Currents were acquired at 20–50 kHz and low-pass filtered at 5 kHz using an analog-to-digital interface (Digidata 1440A acquisition system, Molecular Devices, San Jose, CA, USA). The series resistance was compensated at 75–80%. All pulse protocols were applied more than 5 min after membrane rupture. APs were elicited at 1 Hz by 3 ms, 1.2× threshold current pulses through the patch pipette.

### 2.13. Patch-Clamp Data Analyses

Pulse protocols were applied with a holding potential of −100 mV. Current–voltage (I–V) curves were assessed by depolarizing voltage steps from −80 to 60 mV during 40 ms in 5 mV increments at 1 Hz frequency. Current densities at each test potential were obtained by dividing the INa by cell capacitance. Cell capacitance values did not differ significantly between experimental groups. The AP amplitude (APA) and AP duration (APD) at 30, 70 and 90% repolarization (APD30, APD70 and APD90, respectively) were analyzed using IgorPro8 (ver.8.01) software (WaveMetrics, Portland, OR, USA).

### 2.14. RNA Sequencing and Bioinformatics Analysis

RNA sequencing libraries were prepared using the TruSeq Stranded mRNA kit (Illumina, San Diego, CA, USA) following the manufacturer’s guidelines. The libraries’ quantity was assessed using the 4150 TapeStation system (Agilent, Santa Clara, CA, USA) with High Sensitivity DNA ScreenTape Analysis. Subsequently, sequencing was carried out on the Illumina NextSeq 2000 (100 cycles). The quality assessment of the raw reads was evaluated using the FastQC tool (v0.11.9), and the fastp program (v0.12.4) was used to remove overrepresented polyG sequences. Following this, the reads were aligned to the human genome using the STAR aligner (v2.7.9) with the GRCh38.p13 reference genome and the GENCODE annotation (gencode.v41.primary assembly). Mapped reads were counted with the featureCounts program (v2.0.3). The analysis was performed using three technical replicate samples from two differentiations of one cell line. The identification of differentially expressed genes (DEGs) was carried out using the RStudio package DESeq2 (v1.40.1). The *p*-values of the genes were adjusted with the Benjamini–Hochberg procedure and filtered with p.adj < 0.05; only genes with a |log2 fold change| ≥ 1.5 were considered as differentially expressed. Subsequently, Gene Set Enrichment Analysis and GO Enrichment Analysis of gene sets were performed to obtain signaling pathways using Gene Ontology database.

### 2.15. Statistics

At least three independent experiments (independent cardiac differentiations) were performed for each measurement. Statistical analysis was performed using GraphPad Prism version 5.00 for Windows. Data normality was evaluated by Kolmogorov–Smirnov (n > 50) or Shapiro–Wilk (n < 50) tests. Normally distributed data were compared with a *t*-test; otherwise, the Mann–Whitney test (n < 50) or the Kolmogorov–Smirnov test (n > 50) was applied. An alpha level of 0.05 was used for all statistical analyses. Data are presented as mean ± SD or mean ± SEM.

## 3. Results

### 3.1. Structural Analysis of MYPN-N989I Variant

**Figure 1:**
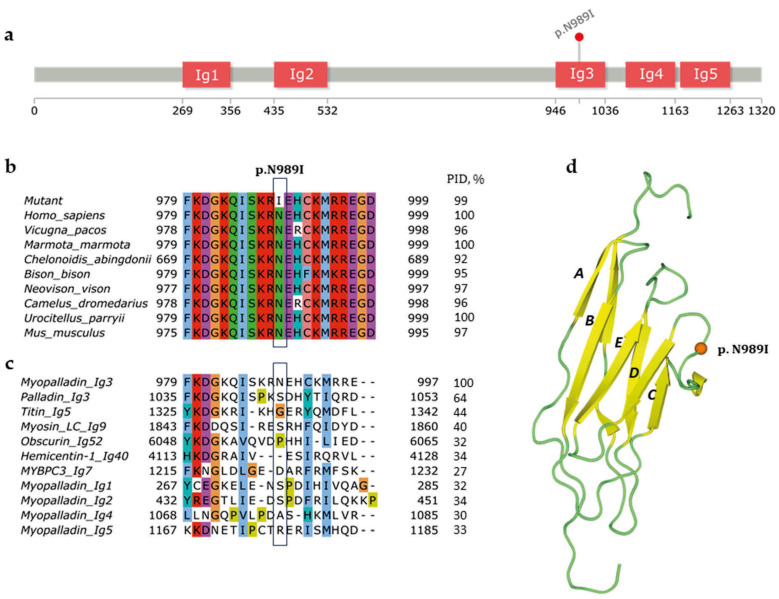
Localization of p.N989I variant in immunoglobulin-like domain 3 of myopalladin. (**a**) Domain organization of myopalladin (UniprotKB: Q86TC9) and localization of the p.N989I variant were performed using the InterPro database [23] and lollipops tool [24]. Immunoglobulin-like domains are marked as ‘Ig’. p.N989I fell in the Ig3 domain of myopalladin. (**b**) A multiple-sequence alignment of human myopalladin with orthologues. (**c**) A multiple-sequence alignment of the Ig3 domain of human myopalladin with Ig-domains of homologous human proteins. p.N989I is located at a variable position within the alignment of the domains. Each residue in the alignment is colored using Clustal X Color Scheme: blue—hydrophobic; red—positively charged; magenta—negatively charged; green—polar; pink—cysteines; orange—glycines; yellow—prolines; cyan—aromatic; white—unconserved; PID—percentage identity performs sequence similarity of Ig domains. (**d**) 3D structure of palladin Ig3 domain (PDB:2LQR) [28]. p.N989I is mapped according to the sequence alignment. A, B, C, D, E—correspond to Ig strands. Substitution fell in the linker (green) between C and D strands (yellow).

A new p.N989I (c.2966A>T) variant of *MYPN* was previously described in a patient with HCM and no family history of inherited cardiac disorders [11]. Myopalladin contains five immunoglobulin-like domains. Structural modeling identified that this variant is located in the third Ig domain of myopalladin (Figure 1a). This domain is known as an α-actinin-interacting domain and contributes to direct F-actin binding, as well as interaction with MRTF-A and regulation of SRF signaling [3]. The variant resides in a highly conserved position among different species (Figure 1b). Alignment of the myopalladin Ig3 domain with homologous Ig domains shows low similarity at this position (Figure 1c). Myopalladin Ig3 is rather similar to palladin Ig3 domain and consists of seven antiparallel β-strands forming a barrel-like shape (Figure 1c,d) [28]. Within the Ig structure, the variant falls in the loop between C and D β-strands (Figure 1d; PDB: 2LQR, chain A). Moreover, ClinPred and AlphaMissense tools predict the p.N989I variant as pathogenic.

### 3.2. Generation of MYPN-N989I iPSC Lines and Directed Differentiation into Cardiomyocytes

**Figure 2:**
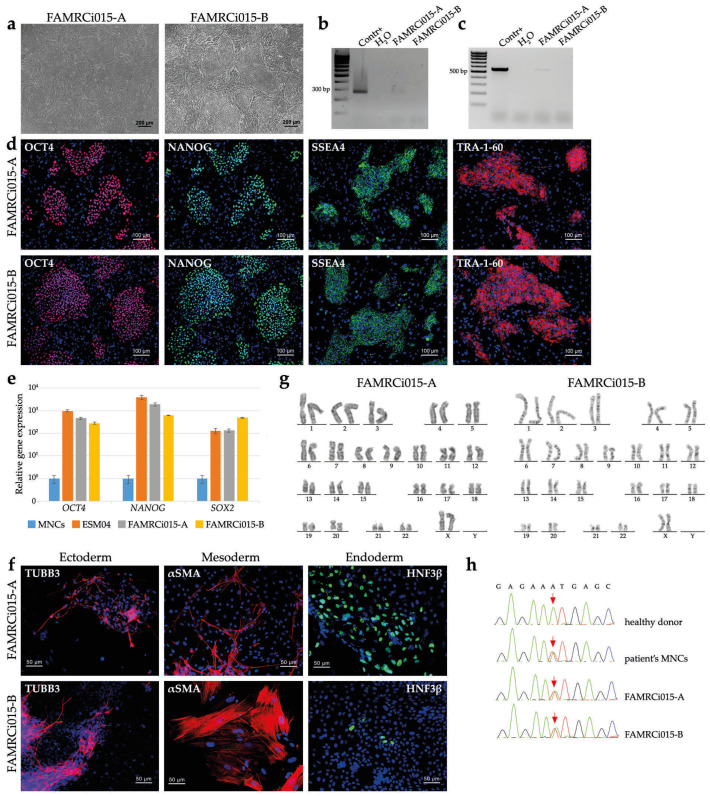
Characterization of iPSC lines generated by reprogramming of the mononuclear cells from the patient with p.N989I (c.2966A>T) variant of *MYPN*. (**a**) FAMRCi015-A and FAMRCi015-B iPSC lines have a morphology characteristic of human pluripotent stem cells. Scale bar—200 µm. (**b**) Absence of mycoplasma contamination in the patient-specific iPSC lines. Contr+, positive control for mycoplasma contamination. H2O, negative control. (**c**) Episome elimination in the FAMRCi015-A and FAMRCi015-B iPSC lines. Contr+, positive control for episome presence, an iPSC line at an early passage. H2O, negative control. (**d**) Patient-specific iPSC lines express a number of pluripotent state markers—the OCT4 and NANOG transcription factors and SSEA4 and TRA-1-60 surface antigens. Scale bar—100 µm. (**e**) Positive expression of pluripotency genes, *OCT4*, *NANOG* and *SOX2*, in the FAMRCi015-A and FAMRCi015-B iPSC lines. MNCs, patient’s mononuclear cells. ESM04, a line of human embryonic stem cells used as a positive control of *OCT4*, *NANOG* and *SOX2* expression. Data are presented as mean ± SD, n = 3 (cell wells for each cell line). (**f**) Patient-specific iPSC lines were able to be differentiated into derivatives of three germ layers—ectoderm (TUBB3, βIII-tubulin), mesoderm (αSMA, smooth muscle alpha-actinin) and endoderm (HNF3β, hepatocyte nuclear factor 3 beta). Scale bar—50 µm. (**g**) FAMRCi015-A and FAMRCi015-B iPSC lines have a normal karyotype—46,XX. (**h**) iPSC lines retain the patient-specific heterozygous p.N989I (c.2966A>T) substitution. The nucleotide sequences of a part of *MYPN* exon 14 in a healthy donor and the patient’s mononuclear cells (MNCs) are given for comparison. The position where the c.2966A>T substitution occurs in the HCM patient is indicated by red arrows.

Mononuclear cells (MNCs) of the patient carrying a heterozygous p.N989I (c.2966A>T) variant of *MYPN* were reprogrammed to the pluripotent state using transfection with episomal vectors expressing *OCT4*, *SOX2*, *KLF4*, *L-MYC* and *LIN28* [35]. The cell lines generated demonstrated human pluripotent stem cell-like morphology (Figure 2a) and were negative for mycoplasma presence (Figure 2b). Based on analysis of episome elimination (Figure 2c), two cell lines, FAMRCi015-A and FAMRCi015-B, were selected for further characterization. The pluripotent state of the cell lines was confirmed by the expression of pluripotency markers (the *OCT4*, *NANOG*, *SOX2* genes and SSEA4, TRA-1-60 surface antigens) and capacity to give rise to derivatives of three germ layers during spontaneous differentiation in embryoid bodies (Figure 2d–f). The iPSC lines retained a normal karyotype, 46,XX, (Figure 2g) and the p.N989I (c.2966A>T) variant of *MYPN* (Figure 2h). STR analysis showed that the iPSC lines were identical to the MNCs used for their generation at 26 polymorphic loci.

**Figure 3:**
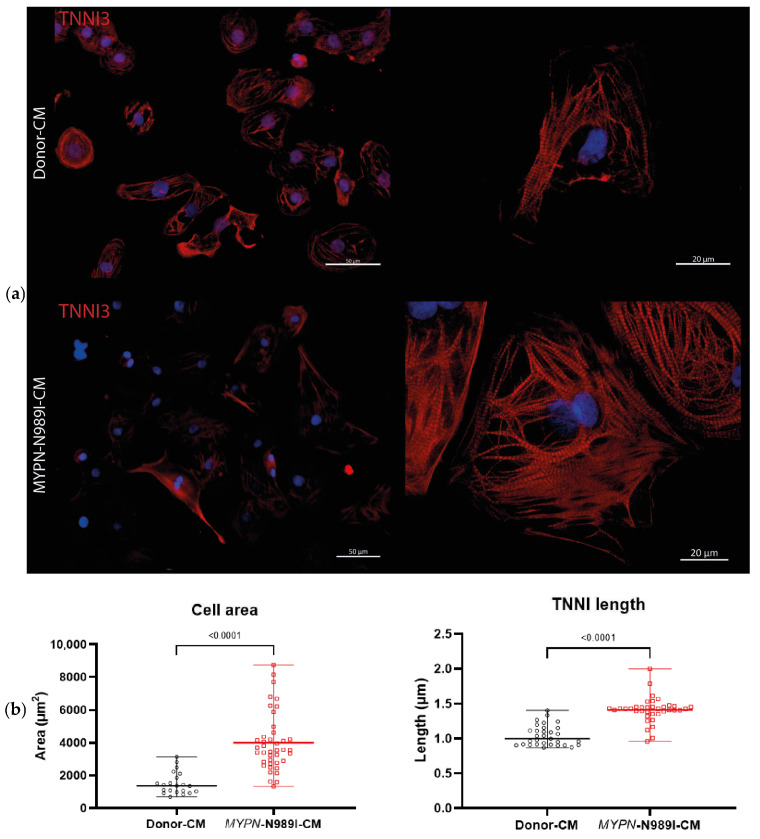
Cell area and sarcomeric length in *MYPN*-N989I-CMs and Donor-CMs. (**a**) Cell area in *MYPN*-N989I-CMs (FAMRCi015-A and FAMRCi015-B iPSC -derived lines) compared to control donor CMs. (**b**) Increased TNNI length in sarcomeric structure of *MYPN*-N989I-CMs (FAMRCi015-A and FAMRCi015-B iPSC-derived lines) in comparison to donor CMs. Data are presented as median ± SD, n > 30.

The patient-specific iPSC lines, FAMRCi015-A and FAMRCi015-B, together with the commercially available iPSC line from a healthy donor, WTSIi046-A (Sigma-Aldrich, Darmstadt, Germany, Cat # 66540120), were differentiated into cardiomyocytes. The protocol based on Wnt activation with the GSK3β protein kinase inhibitor (CHIR99021) followed by Wnt inhibition with IWR1 was used first [36]. Zones of spontaneous contractions formed on days 8–9 of differentiation. To isolate a pure population of cardiomyocytes, the differentiated cells were subjected, from day 9 to day 15 of differentiation, to metabolic selection in glucose-free medium supplemented with sodium DL-lactate [36]. The purified cardiomyocytes were cultivated for two weeks in the presence of 2 µM CHIR99021 for their proliferation and for another two weeks in the medium containing low glucose and high oxidative-substrate concentrations [33] to promote maturation of iPSC-derived cardiomyocytes. The fact that the cells obtained were predominantly cardiomyocytes was verified by expression of cardiac troponin I on day 48 of differentiation (Figure S1). After differentiation was completed, all cell lines demonstrated a cross-sectional pattern and positive staining for cardiac-specific markers. However, in *MYPN*-N989I-CMs, we revealed increased total cardiomyocyte area and enlarged sarcomeric length, as detected by TNNI staining in comparison to Donor-CMs (Figure 3). iPSC-derived cardiomyocytes from the patient with the p.N989I (c.2966A>T) variant of *MYPN* (*MYPN*-N989I-CMs) and the healthy donor (Donor-CMs) were used for functional studies and transcriptome analysis to study the impact of the p.N989I (c.2966A>T) variant of *MYPN* on cardiomyocyte structure and function.

### 3.3. Calcium Dynamics Is Not Altered in iPSC-Derived Cardiomyocytes with p.N989I Variant of MYPN

**Figure 4:**
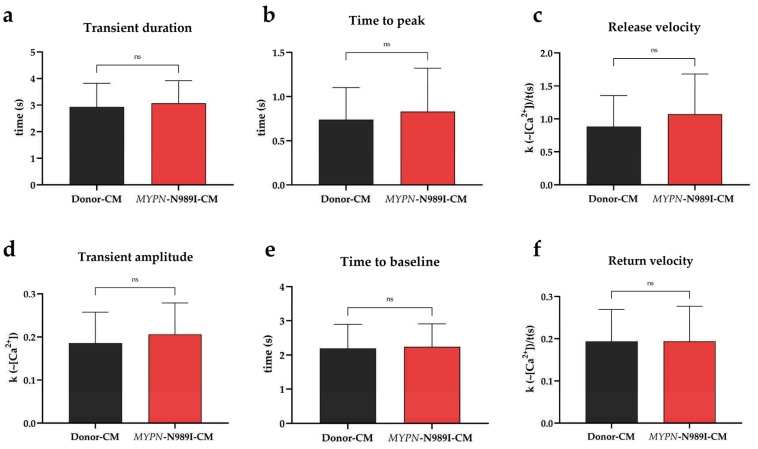
Parameters of calcium transients in iPSC-derived cardiomyocytes of the HCM patient and healthy donor cells. Black columns—healthy donor CMs (one control cell line, two differentiations, total n > 60 technical replicates); red columns—*MYPN*-N989I-CMs (two patient cell lines, two differentiations, n > 90 technical replicates). (**a**) Duration of calcium transient; (**b**) time to transient’s peak; (**c**) calcium rise velocity; (**d**) amplitude of calcium transient; (**e**) time from peak to baseline; (**f**) calcium decay velocity. *p*-value: ns—not significant. The data are presented as mean ± SD.

MyoPacer field stimulation and IonOptix setup were used to measure parameters of calcium transient in the iPSC-derived cardiomyocytes. Transient duration (time to 90% baseline), pre-peak parameters (time to peak and calcium release velocity), transient Ca^2+^ amplitude, and post-peak parameters (time to baseline and calcium return velocity) did not differ significantly between *MYPN*-N989I-CMs and Donor-CMs (Figure 4). To sum up, intracellular cytosolic calcium oscillations in *MYPN*-N989I-CMs were not affected.

### 3.4. MYPN-N989I-Cardiomyocytes Do Not Demonstrate Any Significant Changes in Characteristics of Sodium Current and Action Potential

**Figure 5:**
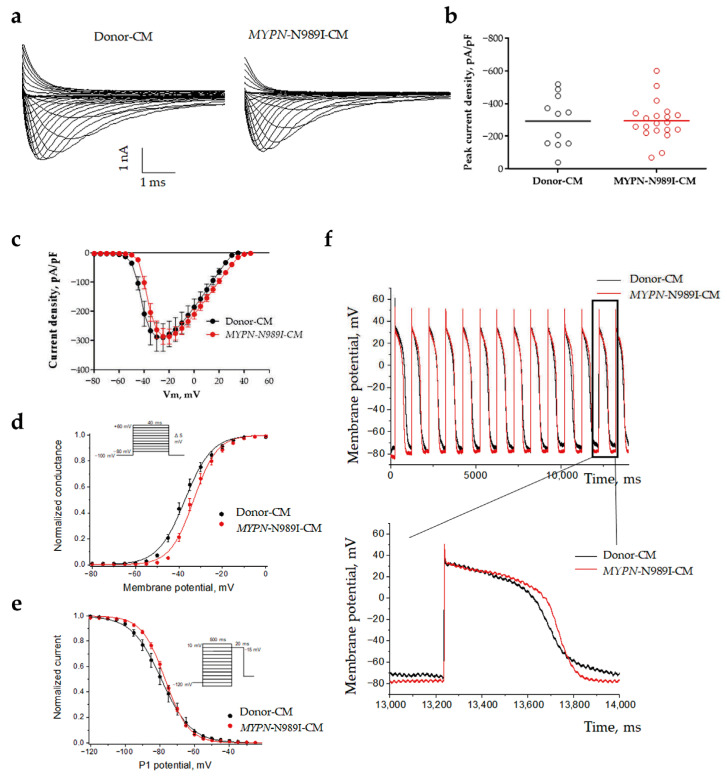
Sodium current parameters and action potential dynamics in iPSC-derived cardiomyocytes of the healthy donor (black, n = 11 technical replicates) and HCM patient (red, n = 20 technical replicates). (**a**) Representative sodium current traces; (**b**) peak current density; (**c**) voltampere characteristics of current density; (**d**) steady-state activation of sodium current; (**e**) steady-state inactivation of sodium current; (**f**) action potential dynamics.

To explore the biophysical characteristics of sodium current in *MYPN*-N989I-CMs, we used the patch-clamp technique. Both Donor-CMs and *MYPN*-N989I-CMs demonstrated typical sodium current traces (Figure 5a). No significant changes were observed in peak sodium current density in *MYPN*-N989I-CMs versus Donor-CMs (Figure 5b,c, Table 1). Next, we analyzed steady-state activation and steady-state inactivation properties (Figure 5d,e, Table 1). We found a statistically significant depolarizing shift of 4 mV in the half-voltage of steady-state activation (Table 1). Thus, *MYPN*-N989I-CMs required more depolarization stimuli to action potential initiation and demonstrated features of sodium channel loss-of-function phenotype. In contrast, no statistically significant alteration of the steady-state inactivation was observed (Figure 5e, Table 1).

We also studied the action potentials in *MYPN*-N989I-CMs and Donor-CMs to clarify the impact of the observed activation shift (Figure 5f). No changes in action potential duration and amplitude were detected (Table 2). Thus, despite the slightly impaired activation in *MYPN*-N989I-CMs, their overall electrical activity demonstrates no alterations.

### 3.5. iPSC-Derived Cardiomyocytes with the p.N989I Variant of MYPN Revealed a Dysregulation of Several Signaling Pathways

**Figure 6:**
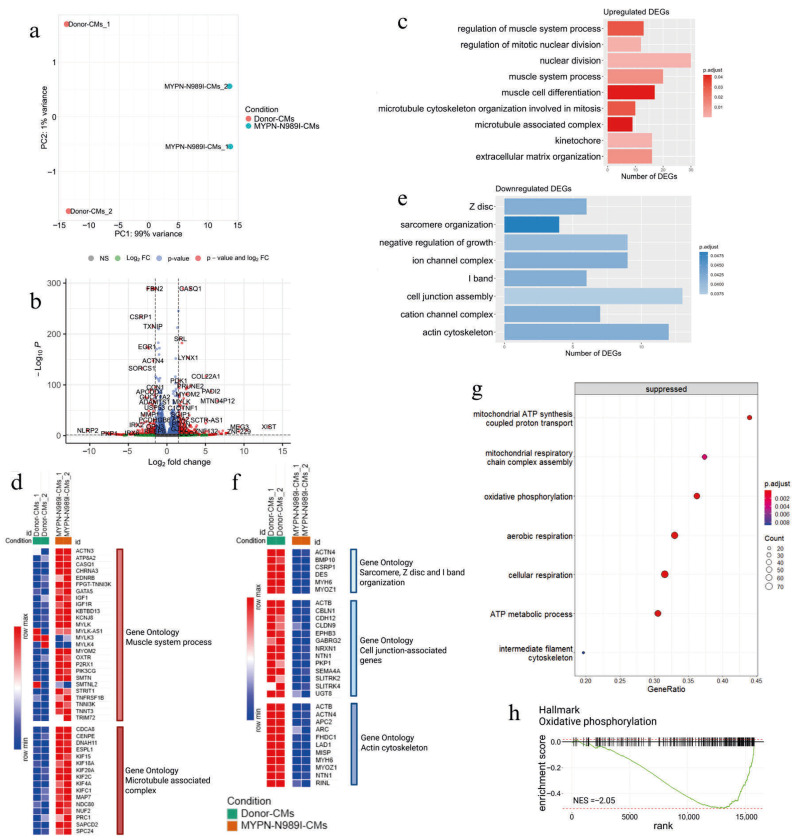
Transcriptome analysis of *MYPN*-N989I-CMs compared to that of Donor-CMs. For control analysis, one control cell line and two differentiations were used; for patient cell analysis, two patient cell lines and two differentiations were used. (**a**) PCA plot; (**b**) volcano-plot of differentially expressed genes (DEGs, |log fold change| > 1.5 and p.adj < 0.05); (**c**) gene set enrichment analysis of upregulated DEGs (p.adj < 0.05); (**d**) heatmap of genes involved in muscle system process and microtubule-associated complex signaling pathways; (**e**) gene set enrichment analysis of downregulated DEGs (p.adj < 0.05); (**f**) heatmap of genes involved in sarcomere, Z-disc and I-band organization, cell junction-associated genes and actin cytoskeleton signaling pathways; (**g**) results of FGSEA; (**h**) GSEA plot of the oxidative phosphorylation signaling pathways from the Hallmark Gene Ontology database (NES = −2.05 and p.adj < 0.05).

To further study the impact of the p.N989I (c.2966A>T) variant of *MYPN* on cardiomyocyte properties, we performed transcriptome analysis of *MYPN*-N989I-CMs and Donor-CMs using RNA sequencing technology. *MYPN*-N989I-CMs and Donor-CMs could be clearly divided by the principal component analysis (Figure 6a). Differential expression analysis revealed 424 significantly upregulated genes (DEGs) and 226 downregulated DEGs in *MYPN*-N989I-CMs compared to Donor-CMs (Figure 6b). To investigate the functional relevance of the detected DEGs, GO analysis using the Gene Ontology database (Biological Process, Cellular Components, Molecular Function) was performed. Significantly upregulated DEGs included genes involved in the regulation of muscle system processes, cell cycle and microtubule cytoskeleton organization (Figure 6c, Table 3). Among genes involved in the muscle system process pathway, the following genes were detected: *CASQ1*, *MYOM2*, *P2RX1*, *MYLK*, *SMTN*, *KCNJ8*, *CHRNA3*, *GATA5*, *TNNT3*, *TNNI3K*, *OXTR*, *KBTBD13*, *ATP8A2*, *PIK3CG*, *TNFRSF1B*, *STRIT1*, *IGF1*, *EDNRB*, *TRIM72*, and *ACTN3* (Figure 6d).

Enrichment analysis revealed upregulation of cell cycle signaling pathways such as nuclear division, regulation of mitotic nuclear division and cell cycle checkpoint. Cyclin-dependent kinase 1 (*CDK1*) was among the genes involved in these pathways—its expression was increased in *MYPN*-N989I-CMs. Interestingly, in *MYPN*-N989I-CMs, we detected a concomitant decrease in the expression of *CDKN1A* (p21) gene—an inhibitor of CDK1. Among other genes linked to the cell cycle signaling pathway, there were genes responsible for the formation of the Ndc80 complex and structuring the mitotic spindle, such as *NDC80*, *NUF2* and *SPC24*. In addition, *MYPN*-N989I-CMs demonstrated activation of genes involved in microtubule–kinetochore conjugation, microtubule assembly, and the microtubule-associated complex, such as *KIF4A*, *NDC80*, *KIFC1*, *NUF2*, *CENPE*, *CDCA8*, *ESPL1*, *KIF15*, *PRC1*, *SAPCD2*, *DNAH11*, *KIF20A*, *KIF4A*, *KIFC1*, *KIF2C*, *KIF18A*, *CDCA8*, *MAP7*, and *KIF15* (Figure 6d, Table 3).

Pathway analysis on downregulated DEGs revealed disturbances in sarcomere organization, Z-disc and I-band organization (*MYH6*, *DES*, *CSRP1*, *ACTN4*, *MYOZ1*, and *BMP10*), cell junction-associated genes (*EPHB3*, *SEMA4A*, *NRXN1*, *GABRG2*, *PKP1*, *CBLN1*, *CDH12*, *SLITRK4*, *SLITRK2*, *UGT8*, and *CLDN9*) and actin cytoskeleton (*ACTB*, *MYH6*, *ACTN4*, *MYO1D*, *LAD1*, *NTN1*, *MYOZ1*, *APC2*, *ARC*, *RINL*, *FHDC1*, and *MISP*) (Figure 6e,f, Table 3).

To further uncover transcriptomic signatures of *MYPN*-N989I-CMs, we also performed a fast gene set enrichment analysis (FGSEA) of non-DEGs using the Gene Ontology database. In addition to the previously described genes, this analysis revealed suppression of signaling pathways associated with ATP synthesis, oxidative phosphorylation and cellular respiration (Figure 6g). FGSEA using the Hallmarks database also revealed downregulation of oxidative phosphorylation (Figure 6h).

**Figure 7:**
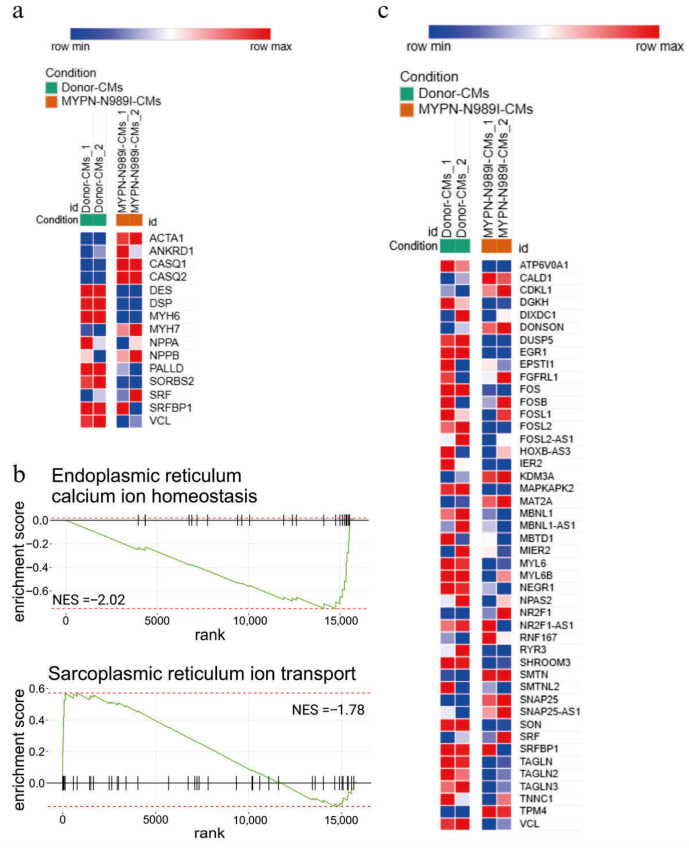
(**a**) Heatmap of Z-disc-associated genes and genes related to Ca^2+^ homeostasis; (**b**) GSEA plot of the endoplasmic and sarcoplasmic reticulum calcium ion homeostasis signaling pathways from the Hallmark Gene Ontology database (NES = −2.02, NES = −1.78 and p.adj < 0.05); (**c**) heatmap of genes involved in the SRF signaling pathway.

To compare the changes observed at the transcriptomic level with earlier published data from other *MYPN*-defective cellular and animal models, we analyzed the expression of several Z-disc-associated genes, genes related to Ca^2+^ homeostasis and genes involved in the SRF signaling pathway [2,3,5]. Decreased expression of *DES*, *DSP*, *VCL*, *PALLD,* and *SORBS2* and an increased *ANKRD1* expression were detected (Figure 7a). We also observed an increased level of *CASQ1* and *CASQ2* in *MYPN*-N989I-CMs compared to in Donor-CMs (Figure 7a). In addition, a marked downregulation of genes related to endoplasmic reticulum and calcium homeostasis, and upregulation of genes related to sarcoplasmic reticulum ion transport were detected, in line with *CASQ1* and *CASQ2* upregulation (Figure 7b). Thus, we were able to confirm changes in the expression level for many earlier reported genes, including those involved in the SRF signaling pathway (Figure 7c).

To sum up, *MYPN*-N989I-CMs demonstrate significant upregulation of myogenic program and activation of cell cycle- and mitotic spindle-linked genes, along with downregulation of sarcomeric, Z-disc- and cell junction-associated genes, as well as ATP synthesis, oxidative phosphorylation-related genes, and genes involved in the SRF signaling pathway.

## 4. Discussion

Mutations in the *MYPN* gene have been demonstrated in connection with various human pathologies. First identified in 2008 in patients with dilated cardiomyopathy, they were later associated with other types of cardiomyopathies, such as hypertrophic and restrictive forms [6,8,37,38]. Along with cardiac disorders, *MYPN* loss of function or homozygous mutations were reported in congenital and slow-progressive myopathies—similar to other genes commonly expressed in cardiac and skeletal muscle. However, interpreting the pathogenic role of MYPN variants remains challenging, especially as they are often detected not in isolation but in combination with other pathogenic variants and do not always clearly segregate with the disease [4,39]. Apart from the causative role of *MYPN* in monogenic cardiac and neuromuscular disorders, this gene was described as one of the genetic loci associated with atrial fibrillation and cardiac remodeling [40,41]. All of the above raise the need for more detailed knowledge on the fine molecular mechanisms of *MYPN*-associated pathologies and the bases for *MYPN*-linked cardiomyocyte dysfunction. The described substitution p.N989I (c.2966A>T) is located in the third Ig domain of MYPN. In a myopalladin homolog, palladin (PLLD), this region is important for direct binding interaction with F-actin [28]. It is possible that substitutions in a similar region of myopalladin can also lead to changes in actinin binding or interfere with protein–protein interactions, leading to functional and adaptive transcriptional changes. To address this question, we created the first iPSC-derived cardiomyocyte cellular model to search for ion channel dysfunction, calcium homeostasis abnormalities and alterations in gene expression profile due to a novel HCM-associated *MYPN*-N989I variant.

MYPN function is mainly demonstrated in connection to the mechanotransduction process within the sarcomeric Z-line and I-band. In tight connection with ANKRD1 (CARP), ANKRD2, TTN, actinin 2, nebulette, and some other Z-line-associated proteins, MYPN facilitates the transformation of mechanical stimuli from the contractile apparatus to biochemical signals and adapts the muscle cells to intrinsic and extracellular stress by activating various stress-response pathways and programs [1,3,42]. The potential interaction of MYPN with PDLIM5, LDB3, OBSCN, and MYOM1 has also been proposed by a computational approach [43]. This tight incorporation into the Z-disc structure makes the protein relevant to the development of protein-surplus skeletal myopathies, such as slowly progressive nemaline myopathies. The mechanism underlying these pathologies is mainly based on the disruption of MYPN interaction with multiple Z-line-associated binding partners, triggering a “domino effect”. In addition to its structural role, a shuttling function between the Z-line and the nucleus in tight interaction with ANKRD2 was demonstrated for MYPN, further supporting its role in mechanotransduction [37,44]. A possible involvement of MYPN in SRF signaling pathways and ERK cascade regulation was also demonstrated by several research groups [3,37]. However, up- and downregulated genes and the signaling pathways activated in response to MYPN mutations are often controversial and depend on the cell line and animal models used and, importantly, the type of mutation and the length of the studied protein [2,3,37]. This may be attributed to the different interaction partners of MYPN within the nucleus and Z-line structure, as well as different effects of loss-of-function and dominant-negative mutations, which lead to completely different molecular mechanisms and clinical phenotypes of MYPN-associated pathologies [4,5,9]. Thus, for example, it is demonstrated that the Y20C-*MYPN* mutation leads to HCM and DCM by a molecular mechanism involving nuclear shuttling and abnormal myogenesis in contrast to the dominant-negative mechanism linked to the Q529X-*MYPN* mutation associated with RCM [5]. In spite of several published studies, no clear functional consequences, apart from the effect on sarcomeric structure and nuclear translocation of MYPN and its partners, have been demonstrated for *MYPN* mutations [2,37].

In our study, we also failed to detect gross functional abnormalities in iPSC-derived cardiomyocytes, either in intracellular calcium dynamics and transients or in voltage-dependent Nav1.5 sodium channel currents. The slight alteration in Nav1.5 kinetics in the form of a depolarizing shift in steady-state activation and features of sodium channel loss-of-function phenotype, however, did not result in significant changes in action potential duration and amplitude, arguing for no remarkable effect of the HCM-associated *MYPN*-N989I variant on overall electrical activity of cardiomyocytes. However, in an earlier study by Filomena et al., the authors demonstrated altered Ca^2+^ handling, namely a delayed Ca^2+^ release and reuptake, as well as reduced Ca^2+^ spark amplitude and the velocity of Ca^2+^ release in *MYPN*-knockout (KO) mice. This difference needs some explanation in terms of MYPN function and the effect on myocyte Ca^2+^ handling. First, it can be attributed to the different systems used in the studies: while Filomena and co-authors used a knockout mouse model and performed Ca^2+^ release studies in the isolated cardiomyocytes with the complete absence of MYPN, we used iPSC-derived cardiomyocytes carrying the heterozygous *MYPN*-N989I substitution. Knowing complex MYPN interactions with structural and regulating proteins, it is reasonable to suggest that one amino acid substitution is not enough to trigger the cascade of reactions leading to alterations in Ca^2+^ homeostasis, while the total absence of MYPN can significantly influence Ca^2+^ handling via abnormal differentiation properties of cardiomyocytes and reduced protein–protein interactions. This mechanism was earlier reported for FLNC-knockout cells and, potentially, can be attributed to many actinin-binding structural proteins [45,46]. In addition, the difference in species-dependent properties—mouse adult cardiomyocytes and iPSC-derived human cardiomyocytes can also explain the observed differences.

In spite of the scarcity of functional changes detected in *MYPN*-N989I-CMs, we were able to detect significant differences in gene expression profile compared to Donor-CMs. With regard to the known limitation of the use of the non-isogenic control cell approach (see the Study Limitations (Section 6) below), significant alterations in pathways and processes associated with the regulation of muscle system processes, differentiation, and cell nuclear division, along with microtubule cytoskeleton organization, correspond to earlier reported data obtained on muscle tissue of *MYPN*-KO mice [3]. This goes along with a downregulation of Z-disc and sarcomere organization and actin cytoskeleton-associated proteins reported by the same authors, further supporting the role of MYPN in driving the myogenic program (Figure 10 from [3]). Of note, in *MYPN*-N989I-CMs, we detected an upregulation of *ANKRD1* similar to that reported earlier by Filomena and co-authors for *MYPN*-KO skeletal muscle cells and cardiomyocytes, as detected by RNA sequencing and real-time PCR [2,3]. A similar observation is valid for *PALLD*, the downregulation of which was registered both in our and previous studies [2,3]. We suggest that the pathogenic effect of the *MYPN*-N989I variant in terms of *ANKRD1* and *PALLD* regulation is similar to MYPN ablation and, possibly, can be linked to the altered protein–protein interactions. Defective interaction of MYPN and ANKRD1 can cause decreased shuttling and translocation of ANKRD1 to the nucleus, thus leading to its upregulation [3,5].

Using RNA sequencing, we also confirmed a downregulation of *DES*, *DSP* and *VCL* in *MYPN*-N989I-CMs—similar to that detected on a protein level in transgenic *MYPN*-Y20C mice and, in addition, demonstrated *SORBS2* downregulation, further underscoring the role of MYPN in Z-disc, desmosome, and cell-contact organization [5]. We managed to confirm the alterations in mitochondrial respiration and ATP production processes in *MYPN*-N989I-CMs—an observation reported earlier at the ultrastructural level in human *MYPN*-Q529X cardiomyocytes [5]. These findings, independently verified in different experimental systems and cell types and for various MYPN mutations, provide strong evidence for MYPN involvement in both Z-disc and cytoskeletal organization, structure and regulation of cellular contacts and mitochondrial function. In addition, in spite of no functional changes in Ca2+ transients, we detected marked derangements in genes related to calcium homeostasis at the transcriptional level. Thus, MYPN-N989I interferes with calcium homeostasis-related genes—an observation aligning with MYPN-KO-related alterations in calcium oscillation in cardiomyocytes [2]. Importantly, we also were able to confirm the downregulation of the SRF pathway in *MYPN*-N989I-CMs—a phenomenon described for *MYPN-*KO skeletal muscle cells [3]—further supporting the role of MYPN in transcriptional regulation, possibly through interaction with the actin cytoskeleton and ANKRD1. Thus, we delineated a dual role of MYPN both in sarcomeric and cytoskeletal organization as well as in muscle program regulation.

## 5. Conclusions

In the current study, we aimed to decipher *MYPN*-associated pathogenetic mechanisms underlying hypertrophic cardiomyopathy. Using the iPSC-technology, we analyzed the functional properties and gene expression patterns in cardiomyocytes carrying a novel p.N989I (c.2966A>T) variant of *MYPN*. Under the described study conditions, we did not detect overt, gross functional abnormalities. Although no significant alterations in parameters of calcium transients, sodium current and action potential were found, the p.N989I (c.2966A>T) variant of *MYPN* had an impact on the transcriptomic profile of iPSC-derived cardiomyocytes. We observed an upregulation of genes linked to the cell cycle, mitotic spindle, microtubule cytoskeleton organization and myogenic program genes, as well as a downregulation of sarcomeric, Z-disc- and cell junction-associated genes. In addition, genes involved in ATP synthesis, oxidative phosphorylation and the SRF signaling pathway were also downregulated. Revealing the deeper links between the observed expression data and a more prominent functional phenotype may require cell-specific stress conditions, detailed immunostaining, electron microscopy studies, or mechanical loading to manifest molecular alterations. However, our results are in good agreement with the previously published data obtained using different cell and animal models and *MYPN* variants. Together, these findings shed more light on the pathogenesis of MYPN-associated cardiomyopathies and myopathies, supporting MYPN’s role not only in sarcomeric and cytoskeletal organization but also in the regulation of the muscle gene program. This information may have additional value in light of newly developed gene therapies and target therapies in the field of cardiomyopathies and neuromuscular disorders, as well as for re-estimating the impact of rare variants of non-sacromeric genes that modify the natural course of genetically determined cardiac pathologies.

## 6. Study Limitations

The study has several important limitations. One of the most significant is the absence of an isogenic control cell line and the use of only one commercial donor iPSC line for control experiments. This negative factor can potentially bias the data obtained, especially the RNA-sequencing analysis, and can, therefore, influence the interpretation of the observed gene expression changes. This issue should be addressed in future experiments by using patient-derived iPSCs carrying the reported MYPN variant along with an isogenic control. Another limitation is the lack of robust protein–protein interaction studies focusing on the Ig3 domain of MYPN and assessing the effect of the MYPN p.N989I variant on actin binding, polymerization, and interaction between MYPN and MRTF-A, as well as on the nuclear versus cytoplasmic localization of MRTF-A. In addition, the non-familial case of HCM and lack of information on family members made a segregation analysis impossible, preventing further assessment of the pathogenic role of this newly described variant.

## Figures and Tables

**Table 1:** Biophysical characteristics of sodium current. Data are represented as mean ± SEM.

		Donor-CMs	n	*MYPN*-N989I-CMs	n	*p*
Current density at −20 mV	pA/pF	−292.1 ± 47.9	11	−294.5 ± 27.0	20	0.9506
Steady-state activation	V_1/2_, mV	−37.1 ± 1.1	11	−33.1 ± 0.9	20	0.0121
	k	5.7 ± 0.3		4.6 ± 0.2		0.0194
Steady-state inactivation	V_1/2_, mV	−79.2 ± 2.0	10	−77.1 ± 0.7	20	0.3012
	k	7.7 ± 0.5		6.5 ± 0.2		0.0114

**Table 2:** Action potential parameters. Data are represented as mean ± SEM.

	Donor-CMs, n = 12	*MYPN*-N989I-CMs, n = 9	*p*
APA, mV	130.2 ± 5.876	125.0 ± 3.314	0.6187
APD90, ms	572.6 ± 52.65	546.8 ± 54.58	0.859
APD70, ms	472.0 ± 47.57	459.5 ± 46.29	0.9717
APD30, ms	338.8 ± 37.13	318.8 ± 42.18	0.594

**Table 3:** Differently expressed genes from the detected signaling pathways.

Signaling Pathway	Gene Symbol	Gene Name	Log Fold Change	p.adj
Muscle system process	*ACTN3*	actinin alpha 3	2.24	3.91 × 10^−2^
	*ATP8A2*	ATPase phospholipid transporting 8A2	1.83	6.62 × 10^−4^
	*CASQ1*	calsequestrin 1	3.06	0
	*CHRNA3*	cholinergic receptor nicotinic alpha 3 subunit	1.66	4.57 × 10^−22^
	*EDNRB*	endothelin receptor type B	4.29	0.01
	*GATA5*	GATA binding protein 5	1.55	1.62 × 10^−8^
	*IGF1*	insulin-like growth factor 1	1.92	6.24 × 10^−3^
	*KBTBD13*	kelch repeat and BTB domain containing 13	2.38	7.47 × 10^−5^
	*KCNJ8*	potassium inwardly rectifying channel subfamily J member 8	1.57	6.09 × 10^−27^
	*MYLK*	myosin light chain kinase	1.90	1.66 × 10^−67^
	*MYOM2*	myomesin 2	2.70	3.46 × 10^−82^
	*OXTR*	oxytocin receptor	2.19	2.75 × 10^−5^
	*P2RX1*	purinergic receptor P2X 1	1.55	9.67 × 10^−81^
	*PIK3CG*	phosphatidylinositol-4,5-bisphosphate 3-kinase catalytic subunit gamma	6.38	1.84 × 10^−4^
	*SMTN*	smoothelin	1.55	3.28 × 10^−40^
	*STRIT1*	small transmembrane regulator of ion transport 1	6.16	4.03 × 10^−3^
	*TNFRSF1B*	TNF receptor superfamily member 1B	1.99	3.96 × 10^−3^
	*TNNI3K*	TNNI3-interacting kinase	1.83	1.03 × 10^−6^
	*TNNT3*	troponin T3, fast skeletal type	3.13	9.81 × 10^−8^
	*TRIM72*	tripartite motif containing 72	1.65	0.02
Microtubule-associated complex	*CDCA8*	cell division cycle associated 8	1.51	2.35 × 10^−6^
	*CENPE*	centromere protein E	1.52	1.21 × 10^−6^
	*DNAH11*	dynein axonemal heavy chain 11	5.04	2.64 × 10^−25^
	*ESPL1*	extra spindle pole bodies like 1, separase	1.68	3.59 × 10^−5^
	*KIF15*	kinesin family member 15	1.64	4.95 × 10^−3^
	*KIF18A*	kinesin family member 18A	2.00	3.19 × 10^−7^
	*KIF20A*	kinesin family member 20A	1.67	1.61 × 10^−15^
	*KIF2C*	kinesin family member 2C	1.71	2.67 × 10^−9^
	*KIF4A*	kinesin family member 4A	1.86	8.12 × 10^−13^
	*KIFC1*	kinesin family member C1	1.80	4.95 × 10^−10^
	*MAP7*	microtubule-associated protein 7	1.59	2.68 × 10^−3^
	*NDC80*	NDC80 kinetochore complex component	2.32	1.50 × 10^−11^
	*NUF2*	NUF2 component of NDC80 kinetochore complex	2.27	1.49 × 10^−9^
	*PRC1*	protein regulator of cytokinesis 1	2.18	1.43 × 10^−2^
	*SAPCD2*	suppressor APC domain containing 2	1.56	0.02
	*SPC24*	kinetochore-associated Ndc80 complex subunit SPC24	1.59	1.06 × 10^−5^
Sarcomere, Z-disc and I-band organization	*ACTN4*	actinin alpha 4	−1.79	4.7 × 10^−148^
	*BMP10*	bone morphogenetic protein 10	−5.37	4.91 × 10^−2^
	*CSRP1*	cysteine and glycine-rich protein 1	−3.40	1.86 × 10^−234^
	*DES*	desmin	−1.51	4.73 × 10^−289^
	*MYH6*	myosin heavy chain 6	−1.95	0
	*MYOZ1*	myozenin 1	−1.68	1.11 × 10^−24^
Cell junction-associated genes	*ACTB*	actin beta	−1.81	0
	*CBLN1*	cerebellin 1 precursor	−1.70	1.83 × 10^−3^
	*CDH12*	cadherin 12	−3.26	5.18 × 10^−3^
	*CLDN9*	claudin 9	−1.66	0.04
	*EPHB3*	EPH receptor B3	−1.56	1.75 × 10^−81^
	*GABRG2*	gamma-aminobutyric acid type A receptor subunit gamma2	−7.74	1.81 × 10^−5^
	*NRXN1*	neurexin 1	−1.71	5.66 × 10^−7^
	*NTN1*	netrin 1	−1.97	3.61 × 10^−63^
	*PKP1*	plakophilin 1	−7.47	5.99 × 10^−5^
	*SEMA4A*	semaphorin 4A	−2.48	3.31 × 10^−12^
	*SLITRK2*	SLIT- and NTRK-like family member 2	−5.83	0.02
	*SLITRK4*	SLIT- and NTRK-like family member 4	−1.58	0.01
	*UGT8*	UDP glycosyltransferase 8	−1.81	0.04
Actin cytoskeleton	*ACTB*	actin beta	−1.81	0
	*ACTN4*	actinin alpha 4	−1.79	4.67 × 10^−148^
	*APC2*	APC regulator of Wnt signaling pathway 2	−3.62	4.09 × 10^−5^
	*ARC*	activity-regulated cytoskeleton-associated protein	−3.93	1.94 × 10^−4^
	*FHDC1*	FH2 domain containing 1	−2.85	0.03
	*LAD1*	ladinin 1	−2.58	6.27 × 10^−75^
	*MISP*	mitotic spindle positioning	−5.56	0.03
	*MYH6*	myosin heavy chain 6	−1.95	0
	*MYO1D*	myosin ID	−2.04	7.09 × 10^−91^
	*MYOZ1*	myozenin 1	−1.68	1.11 × 10^−24^
	*NTN1*	netrin 1	−1.97	3.61 × 10^−63^
	*RINL*	Ras and Rab interactor-like	−1.84	0.02

## Data Availability

The data on the iPSC line characterization are available in the Human Pluripotent Stem Cell Registry (https://hpscreg.eu/cell-line/FAMRCi015-A, https://hpscreg.eu/cell-line/FAMRCi015-B accessed on 12 August 2025). The raw RNA sequence data and counts table were submitted to the GEO database and are available under accession number GSE305865 (https://www.ncbi.nlm.nih.gov/geo/query/acc.cgi?acc=GSE305865, accessed on 19 August 2025).

## Supplementary Materials

The following supporting information can be downloaded at: https://www.mdpi.com/article/10.3390/genes17040456/s1, Figure S1: Cells obtained in the course of directed cardiac differentiation of the patient-specific iPSC lines, FAMRCi015-A and FAMRCi015-B, express a cardiomyocyte marker, cardiac troponin I; Table S1: Primers and antibodies used in this study.
