# Supplementary material for: Functional and Expression Studies of iPSC-Derived Cardiomyocytes Carrying a Novel HCM-Associated MYPN Genetic Variant
Source: Genes (Basel). 2026 Apr 14;17(4):456. doi: 10.3390/genes17040456 (PMC13116294; doi:10.3390/genes17040456)
Supplement: Supplementary file 1 [file genes-17-00456-s001.zip › genes-4175294-supplementary.pdf]

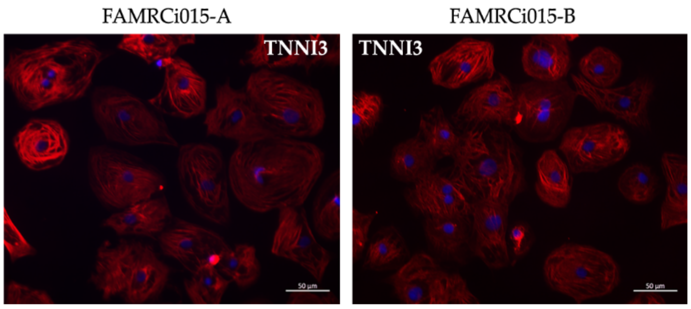

Figure S1. Cells obtained in the course of directed cardiac differentiation of the patient-specific iPSC lines, FAMRCi015-A and FAMRCi015-B, express a cardiomyocyte marker, cardiac troponin I (TNNI3). Scale bar - 50 μm.

Table S1. Primers and antibodies used in this study.

Primers			
	**Gene/Locus**	**Product Size**	**Nucleotide Sequence (5′-3′)**
Mycoplasma detection	16S ribosomal RNA gene	280 bp	GGGAGCAAACAGGATTAGATACCCT/ TGCACCATCTGTCACTCTGTTAACCTC
Episome detection	oriP	544 bp	TTCCACGAGGGTAGTGAACC/ TCGGGGGTGTTAGAGACAAC
Reference gene (RT-qPCR)	*ACTB*	93 bp	GCACAGAGCCTCGCCTT/ GTTGTCGACGACGAGCG
Pluripotency markers (RT-qPCR)	*OCT4*	144 bp	GGGAGATTGATAACTGGTGTGTT/ GTGTATATCCCAGGGTGATCCTC
	NANOG	116 bp	TTTGTGGGCCTGAAGAAAACT/ AGGGCTGTCCTGAATAAGCAG
	SOX2	100 bp	GCTTAGCCTCGTCGATGAAC/ AACCCCAAGATGCACAACTC
p.N989I variant detection in *MYPN*	*MYPN*, exon 14	396 bp	ATGGCAGTTGGCCCTTT/ GTGATGCTGTGCCCTAGTT
**Antibodies**			
	**Antibody**	**Dilution**	**Company, Cat #, and RRID**
Pluripotency markers	Mouse IgG2b anti-OCT3/4	1:50	Santa Cruz Biotechnology, Dallas, TX, USA, Cat # sc-5279
	Rabbit IgG anti-NANOG	1:200	ReproCELL, Yokohama, Japan, Cat # RCAB003P
	Mouse IgG3 anti-SSEA4	1:200	Abcam, Cambridge, UK, Cat # ab16287
	Mouse IgM anti-TRA-1-60	1:200	Abcam, Cambridge, UK, Cat # ab16288
Markers of differentiated derivatives	Mouse IgG2a anti-TUBB3	1:500	BioLegend, San Diego, CA, USA, Cat # 801201
	Mouse IgG2a anti-αSMA	1:100	Dako, Glostrup, Denmark, Cat # M0851
	Mouse IgG1 anti-HNF3β	1:100	Santa Cruz Biotechnology, Dallas, TX, USA, Cat # sc-374376
Cardiomyocyte markers	Mouse IgG2b anti-Troponin I, clone C5	1:200	Sigma-Aldrich, Darmstadt, Germany, Cat # MAB1691
Secondary antibodies	Goat anti-Mouse IgG (H + L) Secondary Antibody, Alexa Fluor 568	1:400	Thermo Fisher Scientific, Waltham, MA, USA, Cat # A11031
	Goat anti-Rabbit IgG (H + L) Highly Cross-Adsorbed Secondary Antibody, Alexa Fluor 488	1:400	Thermo Fisher Scientific, Waltham, MA, USA, Cat # A11008
	Goat anti-Mouse IgG3 Cross-Adsorbed Secondary Antibody, Alexa Fluor 488	1:400	Thermo Fisher Scientific, Waltham, MA, USA, Cat # A21151
	Goat anti-Mouse IgM Heavy Chain Cross-	1:400	Thermo Fisher Scientific, Waltham, MA, USA, Cat # A21043

	Adsorbed Secondary Antibody, Alexa Fluor 568		
	Goat anti-Mouse IgG1 Cross-Adsorbed Secondary Antibody, Alexa Fluor™ 488	1:400	Thermo Fisher Scientific, Waltham, MA, USA, Cat # A21121
	Anti-Mouse IgG BioChemika Antibody, Atto 550 Conjugated	1:500	**Sigma-Aldrich, Darmstadt, Germany, Cat** # 43394
